# Geographic variation in the utilisation of specialist healthcare for patients with severe mental illness in Norway: a population-based registry study

**DOI:** 10.1007/s43999-023-00025-7

**Published:** 2023-06-27

**Authors:** Haji Kedir Bedane, Lars Lien, Maria Holsen, Marte Bale, Knut Ivar Osvoll, Christian Thoresen, Per Arne Holman

**Affiliations:** 1 Research and Innovation Department, Forde Health Trust, Forde, Norway; 2 Norwegian National Advisory Unit on Concurrent Substance Abuse and Mental Health Disorders, Innlandet Hospital Trust, Brumunddal, Norway; 3 Faculty of Health and Social Science, Inland Norway University of Applied Sciences, Elverum, Norway; 4 South-East Regional Health Authority, Oslo, Norway; 5 Department of Patient Safety and Research, Lovisenberg Diaconal Hospital, Oslo, Norway

**Keywords:** Geographic variation, Mental healthcare, Severe mental illness (SMI), Service utilisation

## Abstract

**Purpose:**

The aim of this study is to measure geographic variations in mental healthcare service utilisation among patients with severe mental illness in Norway.

**Method:**

We analysed data from the Norwegian patient registry for 2014–2018 for patients with severe mental illness. The outcomes measured in this study were: outpatient contact, admission, bed days and total contact rates. Total contacts were calculated as the sum of observed outpatient contacts plus four times the hospital bed days for each hospital catchment area based on the Norwegian health director’s report on clinical activity and patient treatment cost. Geographic variations were measured using extreme quotient (EQ), coefficient of variation (CV) and systematic component of variation (SCV). Maps, figures, and tables were used to visualise geographic variation.

**Results:**

The geographic variations saw a six-fold increase in the outpatient contact rate and a three-fold increase in the admission rate between the areas with lowest rate and areas with the highest rate. However, there was low geographic variation in calculated total contact rates (Eqs. _5 − 95 =_1.77). The low-level geographic variation in the total calculated contact rate was also confirmed with an SCV of less than three.

**Conclusion:**

The levels of geographic variations in the utilisation of outpatient and inpatient mental healthcare services among patients with severe mental illness are high. However, the geographic variation in total services provided by hospital catchment areas calculating the two service modalities together using their treatment cost ratio, is low. This may reflect the relatively equal performance of hospital catchment areas in terms of resource utilisation regardless which service modality they prioritise. Factors contributing to high geographic variation in individual service modalities need further investigation.

## Background

Mental and substance abuse disorders are among the largest public health issues in the world, affecting 15% of the global population. In other words, one in seven people are living with one or more mental disorders or substance abuse disorders [1]. Severe mental illness affects approximately 5% of the adult population in the world [2]. Mental disorders can have severe consequences not only for people affected by the disorders, but also for their families and society [3].

In Norway, mental and substance abuse disorders are widespread ,affecting 16–22% of the adult population [4]. Evidence also indicates that mental health disorders affect about 20% of the country’s young male adults and 32% of young female adults [5]. Similarly, one study from urban Norway in 2020 reports that GPs have given a mental health related diagnosis for 19% of their patients aged 16–65 years.

In accordance with the significance of mental health disorders for public health, the government is making efforts to prioritise mental healthcare and to make and implement plans regarding this [6]. During the decade of 2001 to 2009, Norway invested substantially in an action plan to increase and transform mental health services and specialised services in its municipalities [7]. Over the past two decades, the Norwegian mental healthcare system has emphasised the strengthening of its outpatient treatment, and the developing of patient pathways and early intervention, in part as an effort to reduce the need for inpatient treatment. The healthcare system and efforts made by the government are detailed in a recent publication of a healthcare system review [8], and the Norwegian healthcare system has proven to be of a high standard, though it still has some areas for potential improvement [9].

Research on geographical variation in the use of healthcare services has received increasing attention in the last two decades especially, following the pioneering work of Wennberg and Gittelsohn in 1973 [10]. However, studies in geographic variation of mental healthcare utilisation in general and studies involving those with severe mental illness in particular are limited. This limited number of studies has at least been able to document geographic variations in mental healthcare use and further reported various factors related to this, using different measures within mental healthcare [11–16].

The balance of care model, with outpatient and inpatient care, is a major driver of the design and monitoring of Mental Health (MH) ecosystems. It was initially proposed as a framework to balance hospital and community integrated care [17]. According to this framework, services should be available depending on the national income level, but it does not provide any practical suggestions regarding the most appropriate number of services, their capacity, workforce characteristics, and other factors [17]. Length of stay and hospitalisations in inpatient care are lower in MH ecosystems that provide a flexible transition between the two types of services [18]. In one systematic review, the authors have shown that mental health care systems that favour continuity of care approach are associated with a shorter length of hospital stay [19]. A regional Spanish study (2022) found a causal relationship on the number of discharges and length of stay from capacity in outpatient facilities [20].

This study therefore attempts to add to the existing body of knowledge on geographic variation in mental healthcare usage in outpatient visits and hospital admissions on the one hand, and to introduce the “total contact rate” as a measure of mental healthcare utilisation on the other, specifically considering hospital bed days for patients with severe mental illness.

In Norway, recent public reports and findings from the latest health atlas have shown large geographical variations in the use of specialised mental healthcare services [21, 22]. However, none of the previous studies from the Norwegian context considered geographic variation in the total contact rate for patients with severe mental illness, taking both outpatient and inpatient care into consideration.

Thus, the aim of the present study is two-fold. Firstly, it attempts to determine the magnitude of geographical variation in outpatient contact rates, hospital admission and bed day rates for adult patients with severe mental illnesses in Norway. Secondly, it determines the level of geographic variation in total quantity of service delivered using a composite measure, total contact rate (TCR). TCR is calculated as the sum of outpatient contacts plus four times the number of bed days for each hospital catchment area (HCA) in each age and sex category. Thus, we determined an age-sex adjusted TCR for each HCA.

## Methods

### Study setting, population, and period of time

In Norway, specialised healthcare admissions are free at the point of use, despite an out-of-pocket fee for services that are limited to EUR 300 per year for outpatient consultations, equal to EUR 37 per visit in 2022. Services are otherwise funded by general taxation. Four Regional Health Authorities (RHA) are responsible for organising services and setting capacities through a principal agent contract with healthcare trusts. These trusts are responsible for defined hospital catchment areas. Patients are allowed a choice of provider within or across areas [21]. Specialised mental health services are provided by hospitals, community mental health centres (CMHC), and private practitioners contracted by the RHAs. The reimbursement system has a component of activity-based financing for outpatients and there is reimbursement for patient transport.

In the somatic health services, Diagnosis Related Group (DRG) weights for different services is a well-established method to convert consultations and hospital stays into a common currency. Within one healthcare system, the weights are the same. It can be used to measure different activities for reimbursement. In mental healthcare services, few countries have established comprehensive DRGs nationwide. For example, in Norway, this has happened only for outpatient contacts after 2017. The Norwegian finance system is risk-adjusted per capita for need and costs (structure). All four regions use the same financing model. Revenue comes from the general taxation system (85%) and household out-of-pocket payments (15%). Reimbursement for specialist mental healthcare is mostly financed by block grants. For outpatients, 20% of the costs come from activity-based funding [23]. The RHA regulates the capacity, composition of activities and quality through a yearly contract with the trusts. Hence, in most healthcare systems, DRG is not an alternative converter for different activity modalities of mental health services.

In this study, we used data on patient records received for the production of the “Healthcare Atlas for Mental Healthcare and Substance Abuse Treatment” from the national database for specialist healthcare – the Norwegian Patient Register (NPR). This includes the total activity at all institutions between 2014 and 2018 for patients with severe mental illness aged 18 and above. The list of all hospital catchment areas that cover the whole country and the groups of municipalities categorised under each hospital catchment area have been published in earlier health atlas reports [21]. Our aim is not to investigate variation in average cost per patient across regions.

### Study variables, definitions, and sources

Patients with primary or secondary diagnoses (ICD-10 codes) of schizophrenia, schizotypal and delusional disorders (F20–29), mania without psychotic symptoms (F30.1), mania with psychotic symptoms (F30.2), other manic episodes (F30.8), unspecified (F30.9), bipolar affective disorder (F31) or severe depressive episode with psychotic symptoms (F32.3) were considered as patients with severe mental illnesses [21].

We defined outpatient contact as care given to patients in an ambulatory service or outpatient clinic within the same calendar day. We included outpatient contacts that involve the attendance of patients at the point of care in the institution and a stay of less than a day. We defined admission as the care given in inpatient departments with a hospital stay of at least one day at the institution. Admissions of the same patient within eight hours of each other were considered as one admission. Bed days were calculated as the number of days patients stayed at the hospital and is the difference between the date of admission and date of discharge for each patient. TCR is a variable of outpatient contacts and bed days for each HCA in each age and sex category. TCR is adjusted for age and sex. Data on population age and sex were taken from Statistics Norway [24].

### Data processing and analysis

Outpatient contact and admission rates were calculated for each HCA by dividing the number of contacts and admissions by the population of the area, respectively. Rates of bed days were also calculated for HCAs by dividing total bed days by the population. To make rates comparable across HCAs, we standardised rates for age and sex using the direct method of standardisation. We used five age groups for the standardisation with the oldest group being 78 years or older. The Norwegian adult population as of 1st January 2016 was used as the standard population and rates were presented per 1,000 inhabitants.

The Official Norwegian Report – NOU 2019:24 – proposes a new financing model for the four Regional Health Authorities. For adult mental health and addiction services, a weight for consultations is measured as 0.247 of the activity compared to one bed day [25]. A report by the health directorates for the period 2014–2018 reported on the average treatment cost nationwide, where four outpatient contacts are equivalent to one hospitalisation [26]. We therefore argue that “total contact rate” can be used as a proxy for the two activity modalities and converted by the same tariff – 1:4. At an aggregated level and given that the finance system is nationwide, the average costs include a wide variety of different activities. The TCR, constructed based on cost equivalence ratio of the two service modalities (outpatient, inpatient) using actual national data, can be considered as an alternative way to measure the capacity in the two services together.

### Measures of geographic variation used in the study

We described the geographic variations in service utilisation between hospital catchment areas by calculating three descriptive measures. We determined the extreme quotient (EQ) by dividing the highest rate by the lowest rate and calculated the coefficient of variation (CV) by dividing the standard deviation of the rates by the overall average of the rates of all hospital catchment areas [27]. We also calculated the systematic component of variation (SCV), which determines the non-random component of variation in healthcare utilisation rates [27–29]. We recalculated the SCV after excluding the hospital referral areas with the highest and the lowest rates in order to remove the effect of extreme values in the measure of variation. There has been a general suggestion that SCV values above three are likely due to the result of differences in medical practice values, five to ten and greater than ten represent high and extremely high levels of variation between geographic units respectively [27, 30]. We adjusted the SCV for multiple contacts and multiple admissions [31]. To demonstrate the effect of extreme values on measures of variation, we determined the Eqs. _5−95_ and SCV _5−95,_ by excluding the HCAs with a rate below the fifth percentile and above the 95th percentile. It has been documented in a previous study that these measures of variation are affected by extreme values [29].

## Results

Between 2014 and 2018, there was an average of 327,644 outpatient contacts per year across 21,659 individual patients with severe mental illness, equal to 15 contacts per patient per year. Similarly, there were 16,915 admissions and 9,047 individual hospitalised patients per year, leading to an average admission per patient of 1.87 per year. The distribution of outpatient contacts, admissions, length of stay, bed days and total calculated contacts by HCAs are detailed in Table 1.

**Table 1:** Distribution of outpatient contacts, admissions, length of stay, bed days and calculated total contacts and total contact rate per region and hospital catchment area, for adult patients with severe mental illness

Region	HCA^a^	Outpatient contacts	Admissions	LOS^b^	Bed days	CTC^,c^	TCR^d^
South-East	Lovisenberg	28,389	780	28	21,707	115,217	903
West	Stavanger	27,910	2,693	19	50,727	230,818	809
South-East	OUS	22,122	1,192	30	35,400	163,722	753
West	Bergen	36,212	2,591	21	53,368	249,684	699
South-East	Telemark	10,770	984	20	19,756	89,794	688
South-East	Innlandet	19,677	1,559	26	40,522	181,765	624
South-East	Sørlandet	27,188	1,581	19	29,355	144,608	622
North	UNN	9,135	1,398	15	21,002	93,143	622
South-East	Vestfold	13,872	966	24	23,181	106,596	607
North	Finnmark	2,297	456	18	8,304	35,513	599
West	Fonna	11,308	1,100	15	16,582	77,636	564
South-East	Diakonhjemmet	13,219	481	26	12,737	64,167	553
Central	St. Olavs	23,356	1,407	21	29,597	141,744	549
South-East	Østfold	11,180	1,021	26	26,876	118,684	532
North	Helgeland	2,851	269	24	6,552	29,059	494
Central	Møre og Romsdal	14,809	1,144	19	21,241	99,773	488
West	Førde	3,512	397	22	8,816	38,776	482
North	Nordland	6,266	382	27	10,355	47,686	457
Central	Nord-Trøndelag	6,283	435	23	9,840	45,643	457
South-East	Ahus	24,574	1,443	29	41,303	189,786	456
South-East	Vestre Viken	26,818	1,601	21	33,223	159,710	425

^a^Hospital Catchment Area

^b^Length of Stay

^c^Calculated Total Contact = Outpatient contacts + (4 X Bed days)

^d^Total Contact Rate, age and sex adjusted per capita

**Fig. 1:**
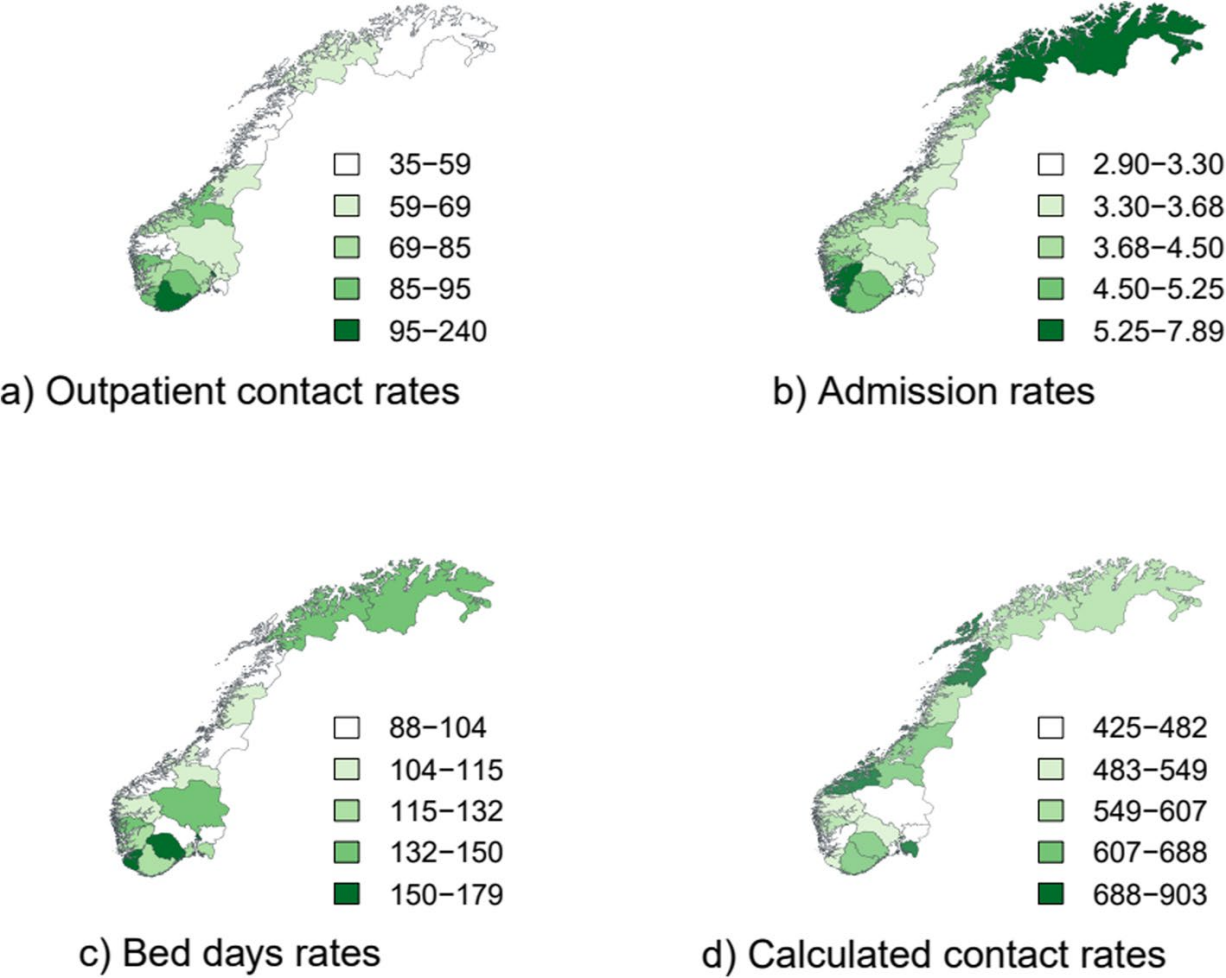
Maps of Norway showing rates of outpatient contacts (**a**), admissions (**b**), bed days (**c**) and calculated total contacts (**d**) per 1,000 inhabitants for patients with severe mental illness by hospital catchment areas in 2014–2018

The catchment areas had different population sizes. The two modalities – outpatient contacts and admissions – obviously varied with size, but not length of stay. Thus, in 2018, the distribution for healthcare contacts ranged from 2,297 to 36,212 for outpatient contacts and 269 to 2,693 for admissions. The number went from 6,552 to 53,368 for bed days and 29,059 to 249,684 for total calculated contacts. Length of stay ranged from 15 to 30 days (Table 2). The geographic variations between HCAs in rates of outpatient contacts, admissions, bed days and calculated total contacts are presented as maps of Norway (Fig. 1). The HCAs in the Northern Regional Health Authority were characterised by lower rates of outpatient contact and higher admission (Fig. 1a and b).

**Table 2:** Hospital Catchment areas’ outpatient contacts, admissions, length of stay, bed days and total calculated contacts for patients with severe mental illnesses, Norway, 2018

Type of contact	Mean	Minimum	Maximum
Outpatient contact	16,273	2,297	36,212
Admissions	1,137	269	2,693
Length of stay (LOS)^a^	22	15	30
Bed days	24,783	6,552	53,368
Total calculated contacts^b^	115,406	29,059	249,684

^a^LOS is bed days divided by admissions

^b^Total calculated contacts is the sum of outpatient contact plus four bed days

**Fig. 2:**
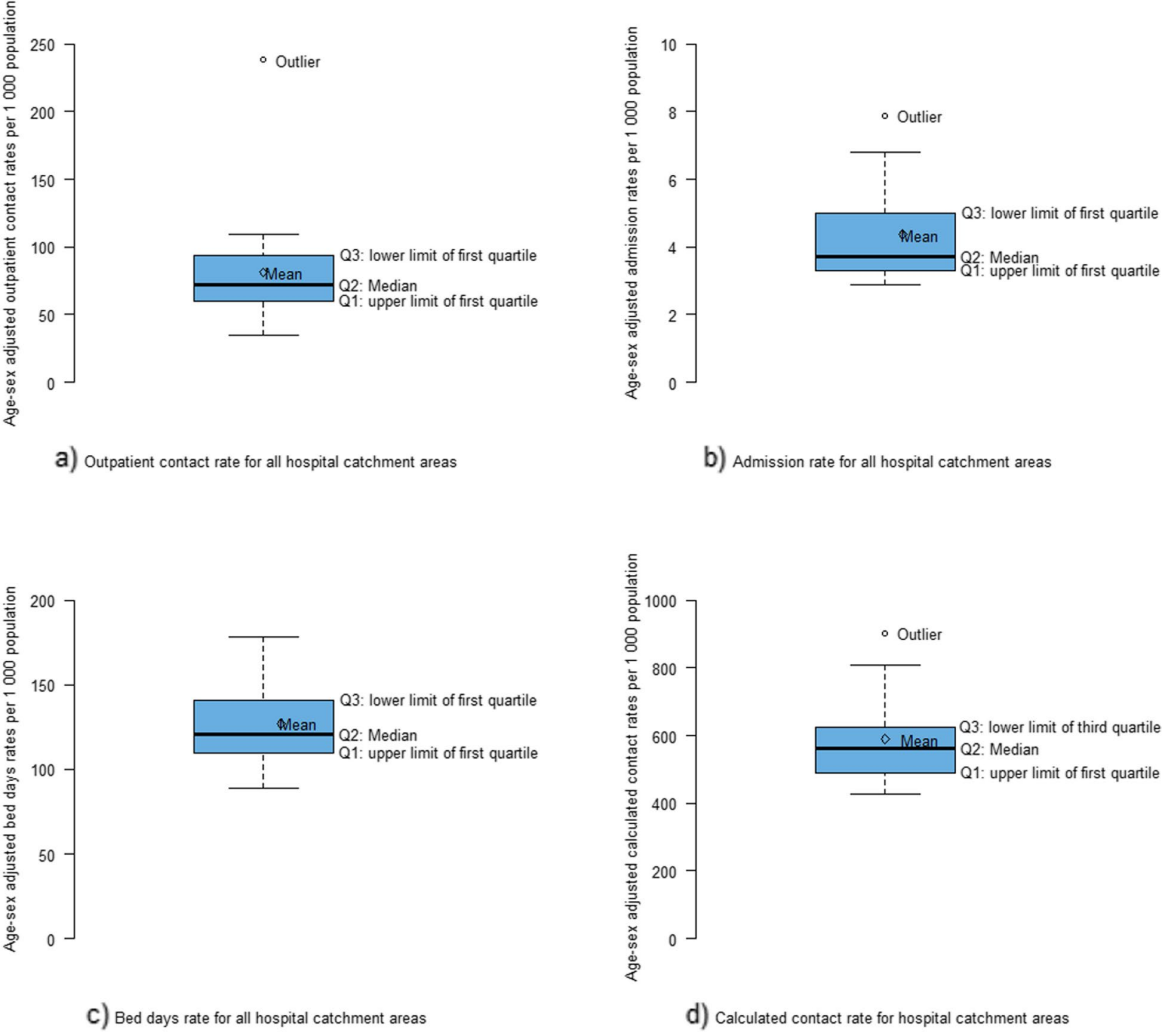
Boxplots of outpatient contact, admission, bed days and calculated contact rates

**Fig. 3:**
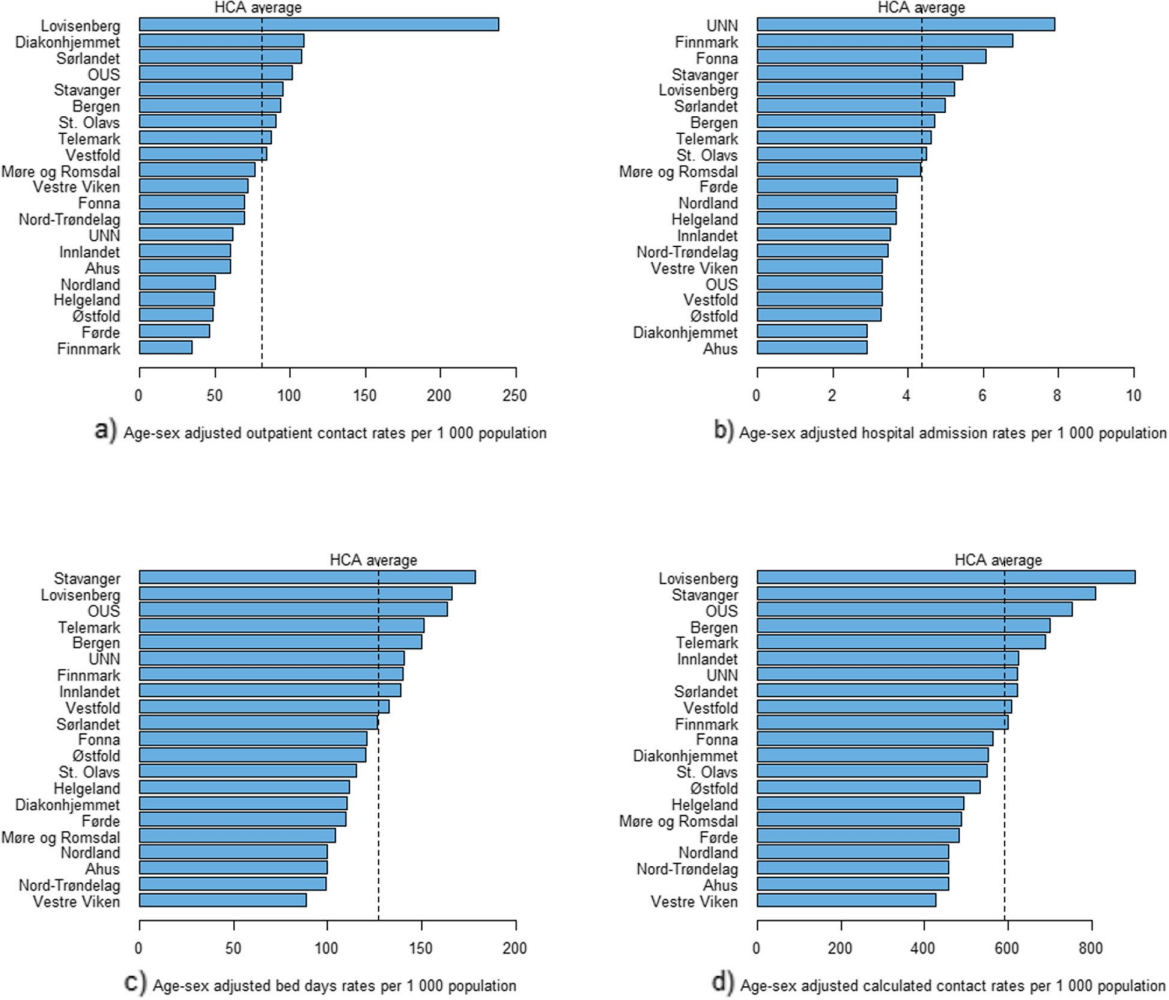
Distribution of rates of healthcare service utilisation in each hospital catchment area, Norway, 2014–2018

The level of geographic variation was high for rates of outpatient contact (SCV_5_-_95_ = 6.46) and admission (SCV_5_-_95_ = 6.11), but low for bed days (SCV_5_-_95_ =2.12) and calculated contact (SCV_5_-_95_ = 2.95) (Table 3). Age and sex adjusted rates per 1,000 inhabitants varied from 35 to 238 for outpatient contacts and from three to eight for admissions. The rate of total calculated contacts per 1,000 inhabitants ranges from 425 to 903. For outpatient contact and admission rates, there was more than a two-fold increase in rates from the area with the lowest to the area with highest, after excluding the areas with rates below the fifth percentile and the area with rates above the 95th percentile (Table 3). A geographical visualisation using boxplots indicated the presence of only one outlying HCA for three of the measures of healthcare utilisation studied (Fig. 2). Similarly, the presence of high levels of variation, apart from the single outlying hospital catchment area, in both outpatient contact and admission rates and low geographic variation in bed days and calculated contact rates were visualised in ordered bar charts that showed the rates in each hospital catchment area for all measures of healthcare utilisation studied (Fig. 3). Even though the overall geographic variation was significantly reduced in total calculated contact rates as compared to the outpatient contact rate and hospital admission, there remains a visible geographical difference between hospital catchment areas with a four-fold increase in total calculated contact rate in Lovisenberg HCA compared to that of Vestre Viken HCA, both located in the South-East region.

**Table 3:** Hospital catchment areas’ variation measures of outpatient contact rates, hospital stay rates and calculated contact rates

Rates per 1,000 inhabitants	Mean	Minimum	Maximum	EQ	Equations _5 − 95_	CV (%)	SCV	SCV_5 − 95_
Outpatient contact rate	81.40	34.97	238.24	6.81	2.33	51.36	14.09	6.46
Admission rate	4.37	2.90	7.89	2.72	2.34	30.50	9.76	6.11
Bed days rate	127.06	88.85	178.66	2.01	1.68	19.69	3.33	2.12
Calculated total contact rate	589.73	425.47	903.13	2.12	1.77	21.32	3.73	2.95

## Discussion

The main finding from this study was that there is a high level of geographic variation in the utilisation of outpatient and inpatient mental healthcare services among patients with severe mental illness. However, there was low geographic variation in capacity, measured by total contact rate that took the two service modalities calculated together, and the geographic variation was observed to be the lowest for bed days rate.

Many countries aim to increase outpatient treatment and ambulatory care for patients with severe mental illness in order to offer better patient pathways and early intervention upon signs of relapse. Although the different levels of treatment are complementary, we argue that it is important to use a measure that integrates several aspects of treating severe mental disorders to better understand and address variations in treatment practices.

The balanced care model for global mental health does not provide any practical suggestions regarding the most appropriate number of services and their capacity [17]. The study from Gipuzkoa in Spain found a causal association between capacities in the two service types [20]. If we assume this effect has relevance for a similar setting in Norway, we suggest TCR to be one alternative method to measure them together. In the Bizkaia Mental Health Services in Spain, Assertive Community Treatment (ACT) teams working with a similar patient group as in our study, reported a decrease in costs from inpatient plus outpatient services by 77%, two years after enrolling in the programme [20]. It has been documented by studies from Norway [32] and Denmark [33] that there is a significant reduction in inpatient service use after the introduction of ACT teams. We therefore argue that one method that takes the cost differences of the two service types into account is useful. Over the last four decades tremendous efforts and investments have been made through national policies and action plans to improve quality and make healthcare equitable across the country [8, 34]. If one evaluates outpatient contacts, admission rates, and bed days as individual measurements, one might misinterpret the level of geographic variations observed. In our study, the variation decreased from 14.09 to 9.97 (SCV) for contact rates and admissions to 3.37 and 2.95 for calculated contact rate (SCV and SCV _5−95_).

The remaining level of geographic variation in total calculated contact rates, particularly between the HCAs of the South-East region of Norway, needs further investigation. Patients’ characteristics related to need and demand for healthcare were not explored in this study, nor were supply-related factors. A public Norwegian report from 2022 documented lower degree of variation in rates of patients with severe mental illness that ranges from 1.2% in Østfold to 2.3% in Finnmark. This finding could be one explanation for the geographic variation in hospital admissions reported in the current study [35]. The four RHAs were reported together, making it impossible to see if the difference in total contact rate in our study could be attributed to the difference in patient rates in the South-East region [35].

The level of variation in total service utilisation, measured by total contact rate reflects that the two service modalities can be complimentary. Furthermore, it may reflect that there is low level of geographic variation between HCAs in use of resources for treating patients with severe mental illness regardless of the type of care. The differences in level of service utilisation between HCAs regarding each type of health service may reflect the difference in choice and priority of Regional Health Authorities and Health Trusts. The magnitude of geographic variation in outpatient contact rates are comparable to what has been reported in a study from England [11]. There are few previous studies that have investigated geographic variation in terms of contact with mental healthcare services in general without specific mental health diagnosis or severity, which makes comparison and interpretation of the results problematic. Furthermore, direct comparison of variations in healthcare between different countries can be problematic due to the varying systems of mental healthcare available in different countries [36].

The level of geographic variation in hospital admissions is lower than what has been reported in one recent study of compulsory psychiatric hospitalisation in Norway [37]. The difference could be attributed to the level of the geographic units and the type of admissions as we assessed variation at the higher geographic unit and included all types of hospitalisation for patients with severe mental illness. Studies from Japan and France have also reported higher geographic variation in general psychiatric inpatient admissions, which could be the result of differences in the patient groups studied, separate from the differences in healthcare systems between countries [38, 39].

Several potential limitations should be taken into account when interpreting and understanding the findings of this study: Our determination of total contact rate based on the cost equivalence between four outpatient contacts and one hospital day considered resource use and aimed to measure the overall quantity of the service used. It should be reported together with outpatient contacts, admissions, and bed days. TCR must not be interpreted as equivalent in quality or intensity of treatments between the two service modalities. Determining equivalence in quality, effectiveness or intensity of the outpatient contacts and hospital bed days is beyond the scope of this study. Furthermore, the difference in the prevalence or severity of illnesses were not considered in our analysis, which might affect observed findings.

Variations in service utilisation might exist at both individual levels and in hospital catchment areas. The current study is limited to the variation that exists between hospital catchment areas and cannot shed light on variations attributed to an individual’s behaviour or characteristics that would then affect their use of mental healthcare services. This implies the need for further studies that include data from the individual level and use of multilevel analysis that breaks down and documents the proportion of variation at different levels. We included face-to-face care and the level of geographic variations reported in this study may be affected by the distribution of other types of mental healthcare services, which do not require patients to be physically present at healthcare institutions. A study including primary care and social services could also investigate the impact of such services on specialised care utilisation.

The current study has some strengths to note. It is based on the national registry covering all areas of Norway. The Norwegian patient registry has proven to be a good source for research data since its establishment [40]. Similarly, the study has made use of the advantage of the good culture of record keeping and reporting of other Norwegian institutions such as Statistics Norway, which operates under a trustworthy data quality control system [41]. We made use of average estimates over a period of five years, which is important for the stability of the results. Moreover, the availability of good registry data enabled the current study to assess geographic variations in both outpatient and inpatient service utilisation and examine the geographic variation for the estimated total service use by combining the two types of services.

In our study, we did not investigate the level of healthcare needed at the level of the HCAs. However, it has been documented by previous reports that there is little or no variation in the distribution of patients with severe mental illness across HCAs, which is one of the measures for healthcare needs [21, 35]. In a recent study of Norwegian mental healthcare services, it was shown that there is still a need to raise the level of care for patients with mental illness [42]. Thus, it could be the under-utilisation of mental healthcare services in some HCAs that has contributed to the huge variations reported in the present study in outpatient contacts and hospital admission rates.

The findings have implications for health authorities, public health practitioners and researchers. Norway is aiming to ensure equal access to a high standard of healthcare for its population. Thus, the geographic variation reported in this study should be considered when revising the national hospital plan’s structure and capacities in mental healthcare services. In light of the limitations of the current study, we recommend further studies that include information at the individual level, which will enable an evaluation of the variation at different levels using multilevel analysis. There is an urgent need for studies that investigate supply and demand related factors contributing to geographic variation in mental healthcare service provision in a Norwegian setting and our team is currently launching a project with this objective.
